# PFN1 and integrin‐β1/mTOR axis involvement in cornea differentiation of fibroblast limbal stem cells

**DOI:** 10.1111/jcmm.14438

**Published:** 2019-09-12

**Authors:** Laura Tomasello, Antonina Coppola, Maria Pitrone, Valentina Failla, Salvatore Cillino, Giuseppe Pizzolanti, Carla Giordano

**Affiliations:** ^1^ Laboratory of Regenerative Medicine “Aldo Galluzzo”, Section of Endocrinology, Diabetology and Metabolism, Department of Promozione della Salute, Materno‐Infantile, di Medicina Interna e Specialistica di Eccellenza “G. D’Alessandro” (ProMISE) University of Palermo Palermo Italy; ^2^ Department of Ophthalmology University of Palermo Palermo Italy

**Keywords:** corneal regeneration, epithelial differentiation, integrin‐β1, limbal stem cells, mTOR pathway, profilin, regenerative medicine, signalling, stem cells

## Abstract

Ex vivo limbal stem cell transplantation is the main therapeutic approach to address a complete and functional re‐epithelialization in corneal blindness, the second most common eye disorder. Although important key points were defined, the molecular mechanisms involved in the epithelial phenotype determination are unclear. Our previous studies have demonstrated the pluripotency and immune‐modulatory of fibroblast limbal stem cells (f‐LSCs), isolated from the corneal *limbus.* We defined a proteomic profile especially enriched in wound healing and cytoskeleton‐remodelling proteins, including Profilin‐1 (PFN1). In this study we postulate that pfn‐1 knock down promotes epithelial lineage by inhibiting the integrin‐β1(CD29)/mTOR pathway and subsequent NANOG down‐expression. We showed that it is possible modulate pfn1 expression levels by treating f‐LSCs with Resveratrol (RSV), a natural compound: pfn1 decline is accompanied with up‐regulation of the specific differentiation epithelial genes pax6 (paired‐box 6), sox17 (sex determining region Y‐box 17) and ΔNp63‐α (p63 splice variant), consistent with drop‐down of the principle stem gene levels. These results contribute to understand the molecular biology of corneal epithelium development and suggest that pfn1 is a potential molecular target for the treatment of corneal blindness based on epithelial cell dysfunction.

## 1 INTRODUCTION

Corneal blindness is the second most common eye disorder and represents a significant clinical problem with many cases of unilateral blindness annually reported and the available cornea for transplantation does not satisfy the surgical demand.^1^ Moreover, several inflammation and stress conditions delay wound healing, leading to epithelial defects and affecting transplant procedure success. Starting from invaluable knowledge about function and regulation of limbal epithelial stem cells, scientists hardly work on both cell therapy and drug therapy to overcome the lack of donor tissues.

To identify new therapeutic targets for improving the regenerative capability of resident or transplanted stem cells, it is important to elucidate about the molecular factors (the *stem triad*, including oct4, sox2 and nanog) and cytoskeleton‐remodelling proteins involved in the regulation of stemness or differentiation decision.^2^, ^3^, ^4^ Several studies have shown that corneal epithelial cell migration involves the integrin‐β1 pathway. By contact with extra cellular matrix (ECM) components, integrin‐β1 transmits signals to influence cell shape, cell proliferation and adhesion, principally modulating the intracellular actin binding‐proteins, including profilin‐1 (PFN1).^5^, ^6^, ^7^, ^8^ A controversial bio‐pathological role was assessed in cancer for PFN1: in breast, pancreas and liver carcinomas it was found down‐expressed, whereas over‐expressed in gastric cancer, in which probably it inhibited proliferation and migration.^9^, ^10^, ^11^, ^12^ In human corneal epithelial regeneration and wound closure involve all limbal stem cell (LSC) populations, among which limbal epithelial stem cells (LESCs) and fibroblast‐like limbal stem cells (f‐LSCs), both hosted in the *limbus*.^13^, ^14^, ^15^, ^16^, ^17^ A conventional therapy to restore the limbal niche consists in transplanting ex vivo expanded LESCs.^18^, ^19^, ^20^ However, this strategy is tainted by several disadvantages such as cell‐passage number limit, allograft rejection risk, the xeno‐feeder proteins and cell contaminations.^21^, ^22^, ^23^ For these reasons, laboratory scientists perpetually evaluated novel isolation and maintenance protocols to improve ex vivo expansion of LSCs. In vitro and in vivo experiments have provided evidence about the biological effects of some natural plant polyphenols, therefore proposed as complement treatment in ocular diseases. In particular, resveratrol (RSV), a grape stilbenoid, revealed a modest beneficial impact on numerous cellular pathways implicated in age‐related ocular disorders, such as oxidative stress, inflammation, mitochondrial dysfunction, apoptosis and survival.^24^, ^25^, ^26^ Recent studies have demonstrated that RSV acts in a dose‐ and time‐dependent manner on MSCs, including human primary keratinocytes.^27^, ^28^, ^29^ In the last few years, our group has focused on the pluripotent and immune‐modulator characteristics in f‐LSCs defined by a proteomic profile especially enriched with remodelling and wound healing proteins, including pfn1 and integrin‐ 1β.^30^, ^31^, ^32^ In the present study, we speculate the existence of a pfn1/integrin‐β1 nanog axis that regulates the stemness/differentiation equilibrium acting with nanog. Moreover, we show RSV treatment being a possible natural drug to modulate this mechanism. Furthermore, we postulate the possibility to obtain epithelial progenitor‐like by f‐LSCs in vitro*.* Taking together, these results suggest pfn1 as a potential molecular target for the treatment of corneal blindness based on epithelial cell dysfunction.

## 2 MATERIALS AND METHODS

### 2.1 Limbus isolation

The study was approved by the Ethics Committee of the AOUP, University of Palermo (No. 09/2009). The informed written consent was obtained by each patient and human tissues were used in accordance with the Declaration of Helsinki. The normal human cornea‐scleral rings from five donors were obtained 2‐3 hours post‐surgery from the Ophthalmology Department (AOUP, University of Palermo). The rings were kept in Hank's Balanced Salt Solution (HBSS, PAA, Pashing, Austria) and then cut into small segments to facilitate separation of the *limbus* from the sclera*.*

### 2.2 Establishment of limbal cell cultures

Human fibroblast mesenchymal stem cells (f‐LSCs) were isolated and cultured as previously described.^31^ Briefly, the limbal segments were incubated with collagenase I overnight, the day after the digest was placed in a dish culture and maintained in DMEM/F12 supplemented with 5% embryonic stem cell‐tested Fetal Bovine Serum (EC‐FBS, PAA, Pashing, Austria), 1X ITS (5 µg/mL Insulin, 5 µg/mL Transferrin, 5 µg/mL Selenium, PAA, Pashing, Austria) and 20 ng/mL basic Fibroblast Growth Factor (b‐FGF, Preprotech, London, UK). Subsequently, the f‐LSCs and LESCs were selected by trypsinization and f‐LSCs subcultures were set up.

### 2.3 Small interfering RNA (siRNA) preparation and cell transfection

Oct4, sox‐2, nanog and profilin‐1 were silenced using appropriate duplex siRNA (the product information and incubation time were reported in Supporting Information 1 (Table 1). The f‐LSC cell transfections were carried out with INTERFERin transfection agent (Polyplus Transfection), according to the manufacturer's instructions. Briefly, cells were seeded into six‐well plates at a density of 2 × 10^4^ cells/well. The transfection agent and siRNA complex were added to the cells and incubated for 72 hours. Each assay was performed in duplicate at least three independent experiments.

### 2.4 Cell viability

Cell viability was evaluated by 3‐(4,5‐dimethylthiazol‐2‐yl)‐2,5‐diphenyltetrazolium bromide (MTT) assay, according to manufacturer's protocol. Briefly, f‐LSCs were grown in 96‐well plates at a density of 2 × 10^4^ cells/cm^2^ and cell viability was evaluated at 24, 48 and 72 hours. The absorbance at 550 nm was determined using MultisKan FC microplate reader (Thermo Fisher Scientific, UK).^33^

### 2.5 RNA extraction, quantification and reverse‐transcription

f‐LSC total RNA was extracted and purified using Rneasy Mini Kit (Qiagen, Milan, Italy) according to the manufacturer's instructions. For quantitative and qualitative analysis Nano Drop 2000 (Thermo Scientific) was used. Total RNA were reverse‐transcribed to cDNA using Reverse Transcriptase Rnase kit (Improm II, Promega, Wisconsin, USA).

### 2.6 Real‐time quantitative PCR (qRT‐PCR)

qRT‐PCR primers were purchased from Qiagen (QuantiTect^®^ Primer Assays, Qiagen, Milan, Italy) and Eurofin MWG (Biotech, Germany) and are listed in Supporting Information 1 (Table 2). All reactions were performed using the Quantitect SYBR Green PCR Kit (Qiagen, California, USA) on the RotorGene Q Instrument (Qiagen, California, USA). Each cDNA sample was mixed with specific primer sets and PCR master mix and amplification were performed as previously described.^31^ Briefly, amplification conditions were the following: 95° for 3 minutes, 40 cycles at 95°C for 20 seconds, 60°C for 30 seconds and 72°C for 60 seconds. Each reaction was performed at least in triplicate. The melting peak analysis determined the specificity of the amplified products. The quantification was performed normalising expression levels to β‐actin‐mRNA levels (used as the housekeeping gene) and comparing to the expression levels of an f‐LSC pool (used as the internal control). The relative gene expression levels were expressed as fold change according to the 2^−ΔΔCt^ method.^34^ The results were represented as histograms on GraphPad Software, Inc, California.

### 2.7 Immunofluorescence staining

The f‐LSC cells were seeded into six‐well plates at a density of 2 × 10^4^ cells/well and the staining was performed as previously described.^31^ Briefly: 1) for cell surface markers the cells were, previously, kept in blocking solution (calcium‐ and magnesium‐free PBS plus FBS10%) for 45 minutes incubated with primary antibody for 30 minutes, and fixed with 2% paraformaldehyde (PFA) for 15 minutes at 4°C; 2) for intracellular cell markers the cells were firstly fixed with 2% PFA for 15 minutes at 4°C, permeabilised with blocking solution added with saponin for 20 minutes and finally stained with primary antibody. The antibody dilution, incubation and detection conditions are shown in Supporting Information 1 (Table 3). Unstained cells were used as a negative control.

### 2.8 Flow cytometry analysis

Cell marker staining was performed using BD Cytofix/Cytoperm™ Plus Fixation/Permeabilization Kit (BD Biosciences, Milan, Italy) according to the manufacturer's instructions. Briefly, 100 µL of cell suspension containing 5 × 10^5^ cells was used for each flow cytometric reaction. The antibody dilution, incubation and detection conditions are shown in Supporting Information 1 (Table 3). All samples were acquired with a FACS Calibur flow cytometer (Becton‐Dickinson, New Jersey, USA) and analysed with the CellQuest Pro software.

### 2.9 Network analysis and GO analyses

Network analysis was performed on the modulated genes coding for the invariant and variant proteins using the STRING (Search Tool for the Retrieval of Interacting Genes/Proteins) website (http://string-db.org/).^35^

### 2.10 Limbal epithelial differentiation

Complete epithelial differentiation medium (EDM) was prepared according to a previously validated composition: low‐glucose Dulbecco's modified Eagle's medium (DMEM) 75% and Ham's F12 medium 25% (both Gibco, UK). This medium was supplemented with the following: foetal bovine serum 10%, hydrocortisone 0.4 µg/mL (PAA Laboratories, Inc, UK) insulin 5 µg/mL (Actrapid®, Novo Nordisk, Denmark), triiodothyronine 1.4 ng/mL (Sigma‐Aldrich, UK), adenine 24 µg/mL (Sigma‐Aldrich, UK), Isoprenaline 4 µg/mL (Sigma‐Aldrich, UK) and EGF 10 ng/mL (PeproTech, Inc, UK).^36^ After 15 days of cultivation in EDM, the differentiation potential was evaluated by qRT‐PCR for CK3 and CK12 the two corneal epithelium‐specific keratins.^37^

### 2.11 Preparation of resveratrol and experimental design

Powder of RSV was dissolved in DMSO as a stock solution at a concentration of 100 mmol/L, and stored at −20°C. The RSV dilution was prepared in a serum‐containing medium at different concentrations (10 μM, 20 μM and 50 μM). Each test was independently run three times with the untreated cells as the control. *Cytotoxicity assay:* the f‐LSCs with or without 10 μM, 20 μM and 50 μM of RSV were seeded in a 96‐well plate at a density of 2 × 10^4^ cells/cm^2^ and cultured up to 72 hours. Cell viability was evaluated by MTT assay.

### 2.12 Protein extraction and Western blot assay

Silenced and un‐silenced f‐LSCs were scraped and incubated in ice for 30 minutes with RIPA buffer (50 mM Tris‐HCl, pH 7.4, 150 mM NaCl, 1% Nonidet P40) and protease inhibitor cocktail (Roche). Total cellular lysate was centrifuged at 14,000 rpm for 1 hour to clear cell debris and the supernatant was stored at −80°C until analysis. Protein concentration in the cellular extracts was determined using Bradford assay. Proteins were denatured in Laemmli sample buffer (2% SDS, 10% glycerol, 5% 2‐mercaptoethanol, 62.5 mM Tris‐HCl pH 6.8, 0.004% bromophenol blue), separated on 12% polyacrylamide gels, transferred to nitrocellulose membranes (TransBlot Transfer Medium Biorad), and blotted with the primary antibodies listed in Supporting Information 1 (Table 3). Antigen‐antibody complexes were visualised using SuperSignal West Femto Maximum Sensitivity Substrate (Pierce) on a CCD camera (Chemidoc, Biorad). Western blot bands were quantified by densitometry using ImageJ software and the results were represented as histograms on GraphPad Software, Inc, California.

### 2.13 Pathway representation

The pathway proposed was represented as a graph on SmartDraw Software, LLC (https://www.smartdraw.com/).

### 2.14 Statistical analysis

All assays were performed in triplicate. The data were reported as means (± standard deviation, SD) and compared using the appropriate version of Student's *t* test. *P values* < 0.05 were considered statistically significant (*P* < 0.05).

## 3 RESULTS

### 3.1 Nanog is the key regulator of stem cell molecular profile in f‐LSCs

**Figure 1:**
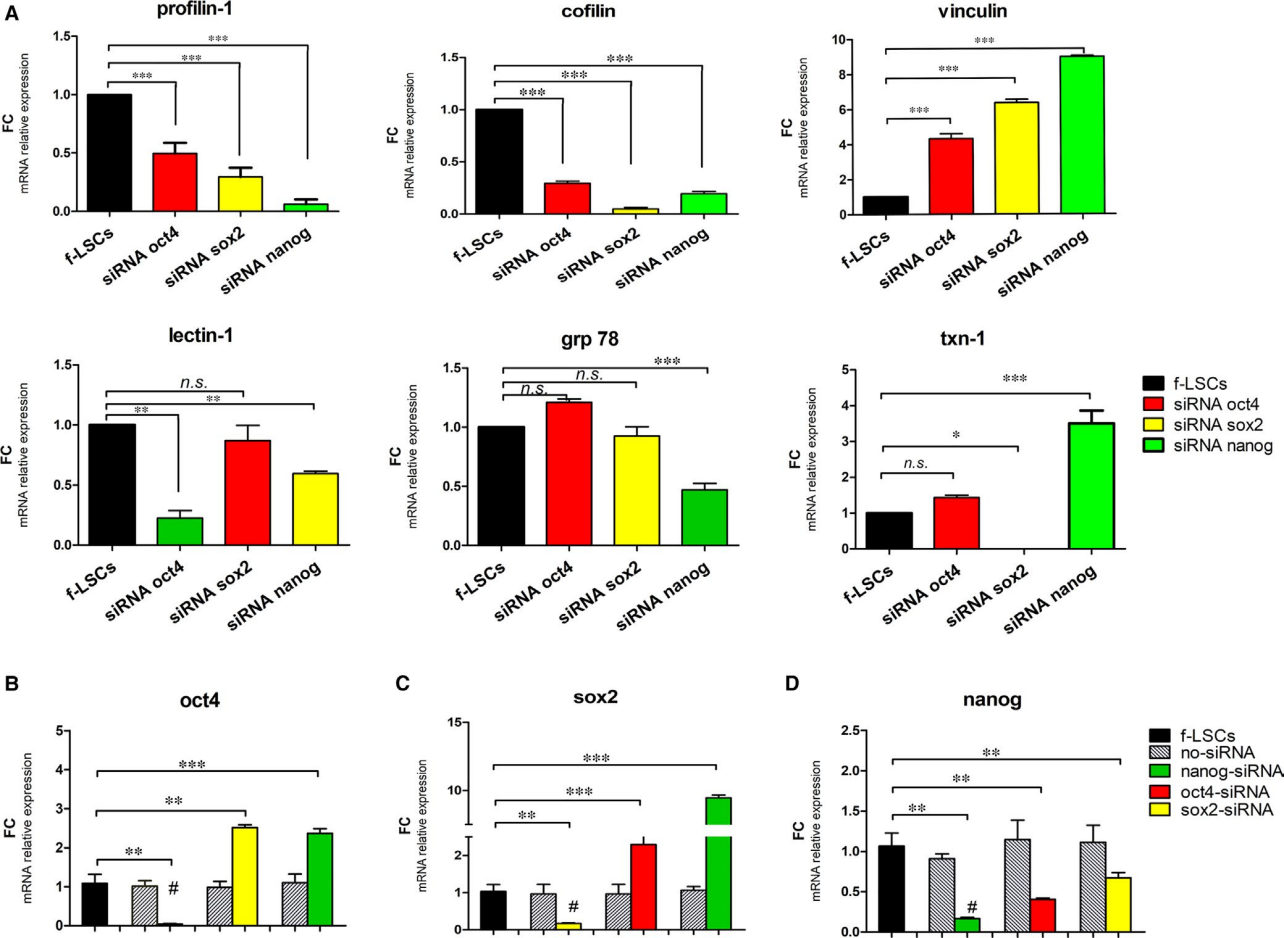
Stem profile after stem transcription factor silencing. A, qRT‐PCR shows the profilin‐1, cofilin, vinculin, lectin‐1, grp78 and thioredoxin‐1 (txn‐1) mRNA levels in oct4, sox2 or nanog siRNA‐transfected f‐LSCs at 72 h vs the no‐target siRNA‐transfected f‐LSCs. B, qRT‐PCR shows the modulation of oct4 mRNA levels in sox2‐ or nanog‐siRNA transfected f‐LSCs vs the control f‐LSCs. C, qRT‐PCR shows the modulation in mRNA level of sox2 in oct4 or nanog‐siRNA transfected f‐LSCs vs the control f‐LSCs. D, qRT‐PCR shows the modulation of nanog mRNA levels in oct4 or sox2‐siRNA transfected f‐LSCs vs the control f‐LSCs. siRNA no‐target f‐LSCs were used as negative controls for all gene silencing experiments. In B, C and D the histogram bars labelled with # represent si‐RNA target gene. The fold change was calculated with the Delta Delta Ct method using the Rotor Gene Q software and the expression was normalised for the housekeeping gene β‐actin. The histogram graphs were performed by GraphPad Software, Inc, California and are represented as ± SD with *P < 0.05, **P < 0.02, ***P < 0.001, *ns=* no significant, SD = Standard deviation; *P* = *P* value, FC = fold change. All pictures are representative of three independent experiments

We previously identified a stem proteomic f‐LSC profile (SPP) by two‐dimensional gel electrophoresis (2D‐IPG) including some wound healing proteins, that is profilin‐1, cofilin, vinculin and grp78, lectin‐1 and thioredoxin‐1 (txn1) (Supporting Information 2, Figure 1). To elucidate the association among the stem transcriptional triad and the stem proteomic f‐LSC profile, several gene‐silencing experiments were performed. The f‐LSCs were incubated with oct‐4, sox2 and nanog siRNA individually up to 72 hours, and finally the mRNA levels of gene coding for the SPP were evaluated (Figure 1A). Relative expression analysis revealed a significant modification in the fold change (FC) values for all stem gene markers (Supporting Information 2, Table 4). In particular, in siRNA‐transfected f‐LSCs significant reduction in the mRNA levels of profilin‐1 and cofilin‐1 was detected, together with an appropriate elevation of vinculin mRNA levels, when compared to the control f‐LSCs. In detail, profilin‐1 mRNA levels were decreased up to 0.49 ± 0.09, 0.29 ± 0.07 and 0.06 ± 0.012 FC in oct4, sox2 and nanog siRNA‐transfected f‐LSCs, respectively (*P* < 0.001) when compared to the no‐target siRNA f‐LSCs.

To understand if a hierarchy exists behind the regulation of the stem transcriptional triad, we observed the behaviour of all three genes after silencing each of them up to 72 hours. The efficiency for gene silencing experiments of oct4, sox2, nanog was 91.51%, 88,72% and 83.32% respectively (represented in Figure 1B,C by # sign). In oct4 siRNA‐transfected f‐LSCs, we found over‐expression of sox2 mRNA levels compared to the control f‐LSCs (up to 2.15 ± 0.86 fc, *P* < 0.001) (Figure 1B), whereas in sox2 siRNA‐transfected f‐LSCs higher oct4 mRNA levels were detected (2.52 ± 0.75 fc, *P* < 0.02) (Figure 1C). It is noteworthy that in nanog siRNA‐transfected f‐LSCs, both oct4 and sox2 mRNA levels were over‐expressed, as is demonstrated by the up‐regulation of 2.37 ± 0.54 FC (*P* < 0.001) in oct4 mRNA levels and 9.45 ± 1.21 FC in sox2 mRNA levels (*P* < 0.001) respect to the control f‐LSCs (Figure 1B,C). Equally in oct4 and sox2 siRNA‐transfected f‐LSCs the nanog mRNA levels were decreased when compared to the control f‐LSCs (0.40 ± 0.08 and 0.58 ± 0.13 FC, *P* < 0.02, respectively) (Figure 1D). These results suggest, first of all, that sox2 expression was maintained at a higher level in each experimental condition*,* and secondly that a compensatory mechanism probably exists for oct4 and sox2 expression and finally that oct4 and sox2 expression is similarly regulated through a possible negative feedback which is nanog‐dependent.

### 3.2 Nanog silencing affects cell cycle promotion and increases expression of important differentiation markers in f‐LSCs

**Figure 2:**
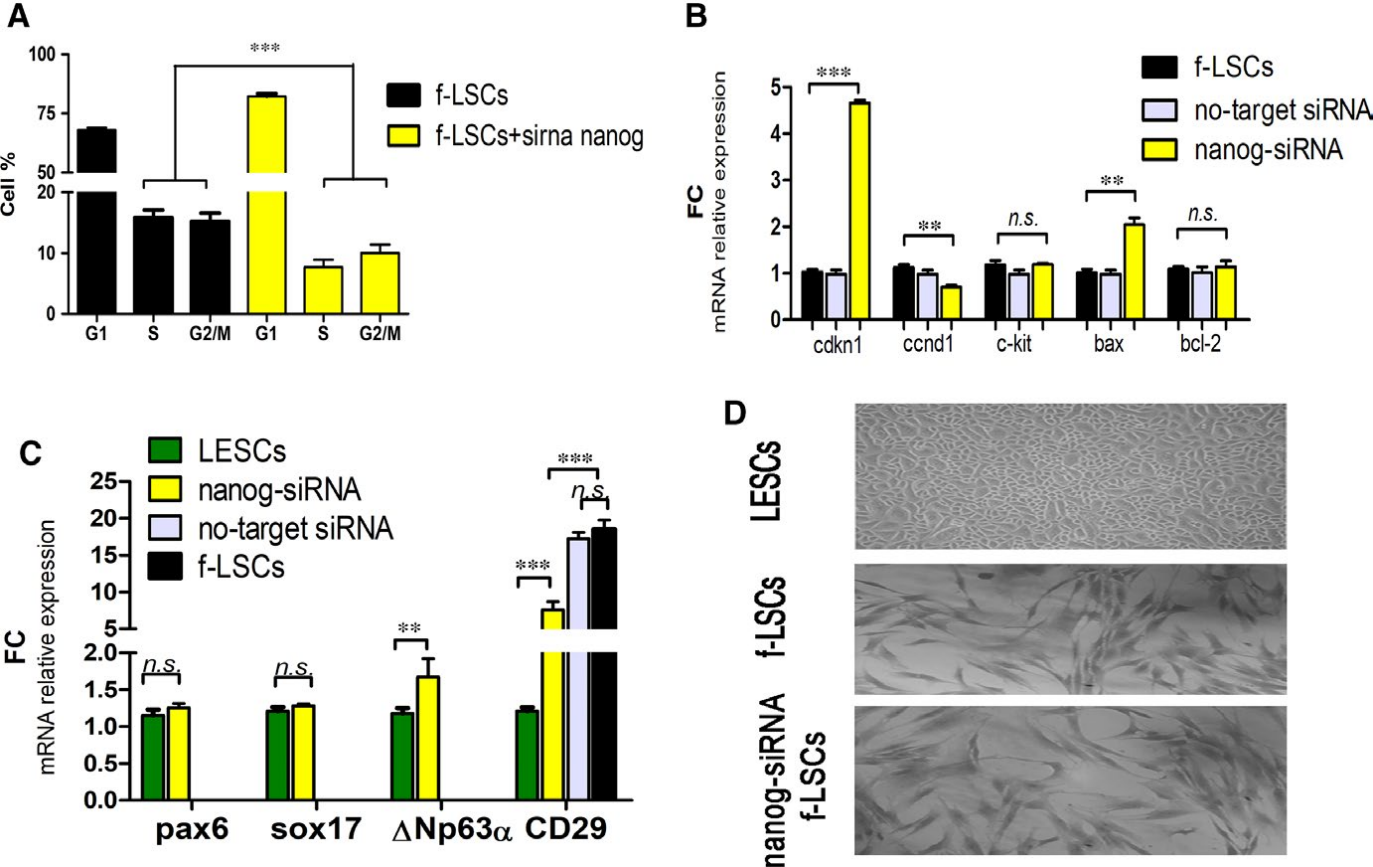
Nanog‐dependent lineage commitment in f‐LSCs. A, The bar plot represents the comparative cell cycle distribution analysis between nanog‐siRNA transfected f‐LSCs and f‐LSCs at 72 h silencing. B, qPCR analysis of cell cycle regulators and proliferation markers cdnk1b, ccnd1, c‐kit, bax and bcl‐2 in nanog‐siRNA transfected f‐LSCs vs the control f‐LSCs, at 72 h silencing. No‐target siRNA f‐LSCs were used as a negative control. C, The bar graphs represent the relative mRNA expression of early epithelial progenitor markers, pax6, sox17 and ΔNp63α, and limbal stromal mesenchymal, CD29 (intregin‐1β), in nanog‐siRNA transfected f‐LSCs vs the control f‐LSCs, at 72 h silencing. LESCs and f‐LSCs were used as positive control of the epithelial progenitor markers and the stromal mesenchymal stem cell markers, respectively. The bar graphs were done using GraphPad Software, Inc, California and are represented as ± SD with **P < 0.02, ***P < 0.001, *ns=* no significant, SD = Standard deviation; *P* = *P* value, FC = fold change. All pictures are representative of three independent experiments. D, Optical microscope images (20x) represent the typical cuboidal cell shape of LESC monolayer subculture (top image), the fibroblast‐like shape of f‐LSCs (middle image) and no difference in morphology in nanog‐siRNA transfected f‐LSCs (bottom image) at 72 h. Photos were capture by Nikon DS‐fI1

The cell cycle analysis was performed by flow cytometry to define if nanog silencing alone negatively affects the cell cycle progression. The f‐LSCs were incubated with nanog‐siRNA up to 72 hours: after silencing, a reduced number of cells in S‐ and G2‐phase and an increased one in G0/G1‐phase indicated cell cycle arrest (Supporting Information 2, Figure 2). The proliferation index (PI) was estimated at 7.69 ± 2.15% and 29.81 ± 4.57% (*P* < 0.02), in nanog siRNA‐transfected f‐LSCs and in the control f‐LSCs, respectively (Figure 2A). Moreover, we evaluated the principal genes involved in cell proliferation and apoptosis regulation, by qPCR analysis: c‐kit (also SCFR, Stem cell factor Receptor), ccnd1 (cyclin‐D1) and cdkn1b (cyclin‐dependent kinase inhibitor 1B) and bax (Bcl‐2‐associated X protein, pro‐apoptotic gene) and bcl‐2 (B‐cell lymphoma 2, anti‐apoptotic gene). A significant down‐expression in ccnd1 mRNA levels was detected (0.75 ± 0.13 FC, *P* < 0.02), whereas no differences in c‐kit and bcl‐2 mRNA levels (*P* > 0.05) were found. Interestingly, significant hyper‐expression of cdkn1b and bax mRNA levels (4.66 ± 0.15 FC, and 2.11 ± 0.53 FC, *P* < 0.001, respectively) in nanog siRNA‐transfected f‐LSCs was found, when compared to the control f‐LSCs (Figure 2B). In light of this, we investigated the presence of epithelial lineage genes: in nanog siRNA‐transfected f‐LSCs mRNA expression levels of pax6 (paired‐box 6) and sox17 (sex determining region Y‐box 17) showed no significant differences compared to LESCs (the positive control). By contrast, significant down‐expression of integrin‐1β (CD29) compared to the control f‐LSCs, was detected (Figure 2C). However, no change in cellular morphology at 72 hours was observed (Figure 2D). Therefore, we concluded that a decreased expression of nanog is accompanied by induction of f‐LSCs towards epithelial lineage.

### 3.3 Limbal epithelial commitment and epithelial differentiation potential of f‐LSCs

**Figure 3:**
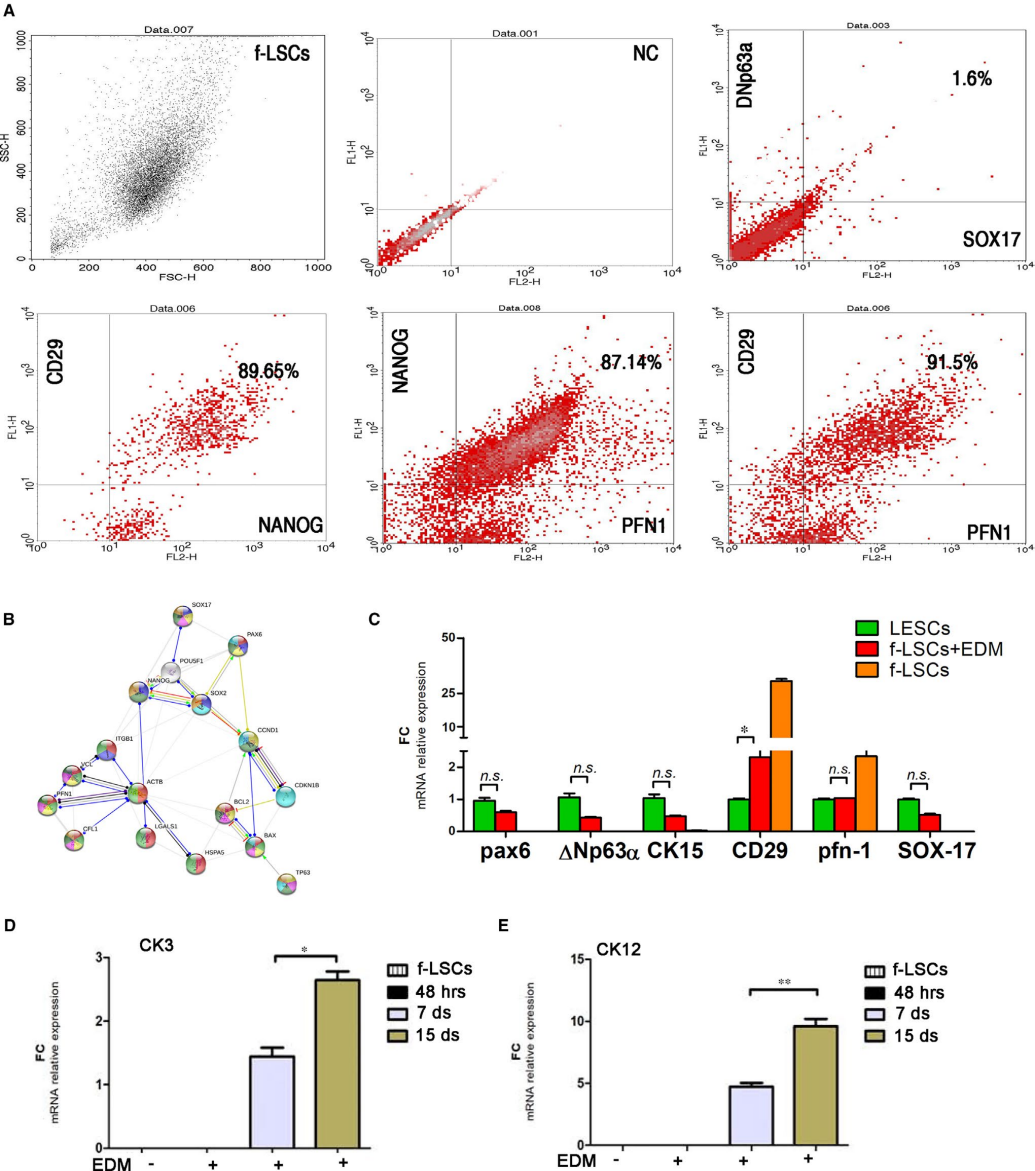
f‐LSC epithelial differentiation potential. A, A representative flow staining of double negative fresh f‐LSCs for ΔNp63α/SOX17 (upper panel), double positive f‐LSC population for PFN1/CD29, PFN1/NANOG and SOX2+/CD29 (in lower panel), NC = Negative Control (Isotype control), CD29 = integrin‐β1. B, The node directed graph represents the protein‐interaction networks analysis that occurs in f‐LSCs highlighting the epithelial differentiation potential. C, The bar graphs represent the relative mRNA expression of epithelial progenitor markers, pax6, ΔNp63α, CK15, sox17 and limbal stromal mesenchymal, CD29, in differentiated f‐LSCs (f‐LSCs + EDM), no treated f‐LSCs and LESCs, (CD29 = integrin‐β1, EDM = epithelial differentiation medium); D, E, The bar graphs represent relative mRNA expression of corneal epithelial cytokeratins, CK3 (D) and CK12 at different time points of differentiation process (48 h, 7 and 15 d) (E). The bar graphs were done using GraphPad Software, Inc, California and are represented as ± SD with *P < 0.05, *ns* = no significant, SD = Standard deviation; *P* = *P* value. All pictures are representative of three independent experiments

**Figure 4:**
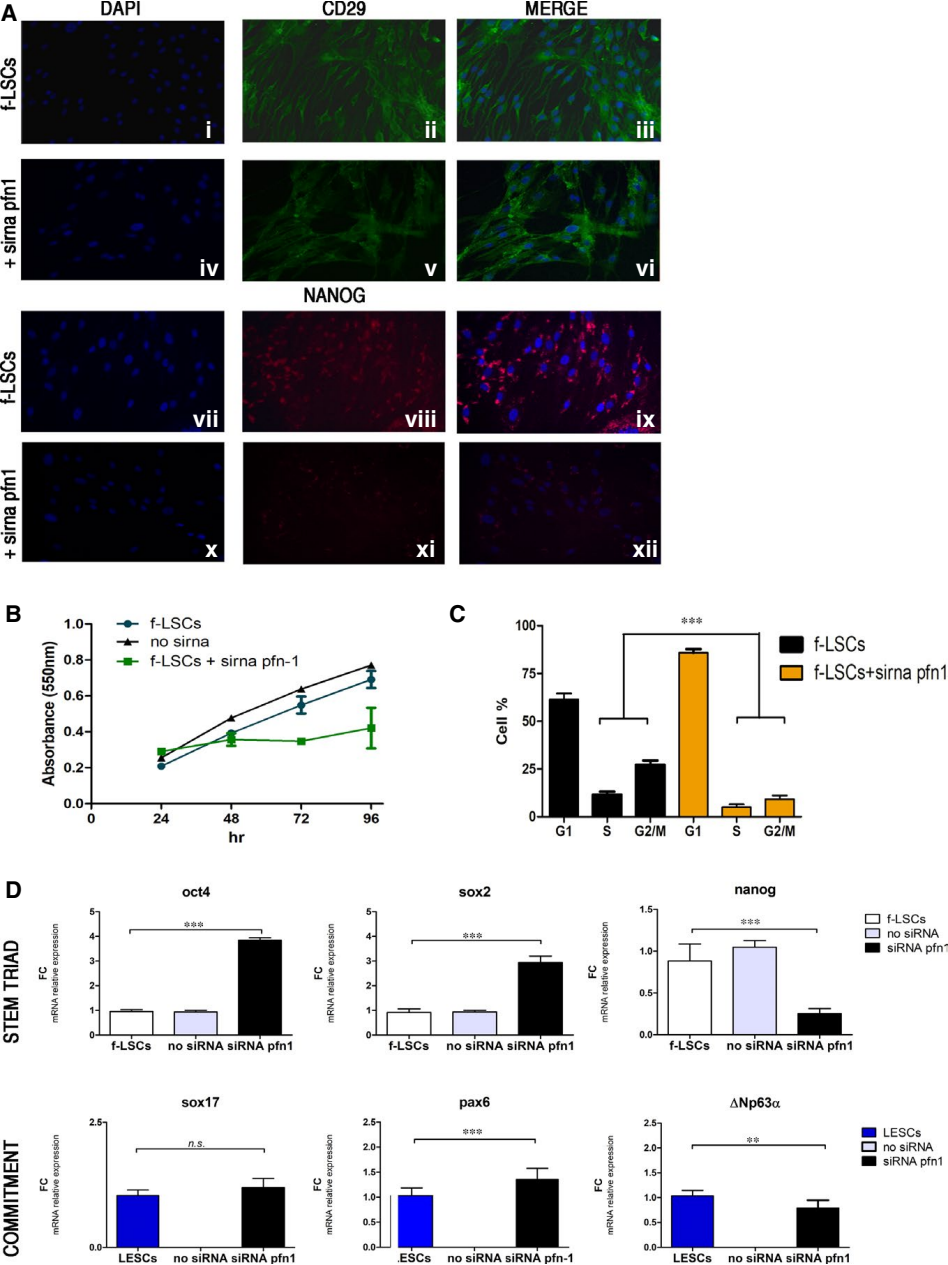
The integrin‐β1, profilin‐1 and nanog axis: pfn1 silencing drives f‐LSCs to the epithelial differentiation. A, Immunofluorescence assay for integrin‐β1 (CD29) and NANOG in no‐siRNA f‐LSCs (i‐iii, vii‐ix) and pfn1‐siRNA transfected f‐LSCs (iv‐vi, x‐xiii). In green and red staining, NANOG and integrin‐β1 respectively, in blue staining the cell nuclei. B, Proliferation assay (MTT) at 96 h for the control f‐LSCs (dark blue), no‐siRNA f‐LSCs (black line) and pfn1‐siRNA transfected f‐LSCs (green line). C, The bar plot represents the comparative cell cycle distribution analysis between pfn1‐siRNA transfected f‐LSCs and the control f‐LSCs (G2M + S = proliferation index (PI)), at 72 h silencing. D, qPCR analysis of stromal mesenchymal stem cell markers oct4, sox2 and nanog (top panel) and early epithelial progenitor markers sox17, pax6 and ΔNp63α (bottom panel) in pfn1‐siRNA transfected f‐LSCs vs the control f‐LSCs and in pfn1‐siRNA transfected f‐LSCs vs LESCs, respectively. The bar graphs were performed by GraphPad Software, Inc, California and are represented as ± SD with **P < 0.02, ***P < 0.001, *ns =* no significant, SD = Standard deviation; *P* = *P* value. All pictures are representative of three independent experiments

The fresh f‐LSCs were run for flow cytometry analysis of PFN1, integrin‐β and NANOG, as mesenchymal stem cell markers, and for ΔNp63α (p63 splice variant) and SOX17, as the epithelial commitment markers. As shown in Figure 3A the f‐LSCs represent a higher double stained cell population for PFN1/integrin‐β1 and PFN1/NANOG (84.7 ± 6.2% and 83.17 ± 5.75%, respectively) and were negative for ΔNp63α and SOX17. Moreover, a protein‐interaction networks (PIN) analysis was performed on https://string-db.org. We involved in the computational exploration the SPP, the stem transcriptional triad and the epithelial related proteins. A more extended search for interaction highlighted the implication of cell cycle gene regulation, that is bax, ccnd1 and cdkn1b and of integrin‐1β (Figure 3B). A significant co‐relation was found between all proteins included in the investigation (*P* < 6‐15): ten of them were proteins of the response to wounding (red nodes in Figure 3B), five proteins were involved in cell fate specification (dark violet nodes in Figure 3B), six proteins in regulation of epithelial cell proliferation (light blue nodes in Figure 3B), eight were related to cell activation (light green nodes in Figure 3B), nine proteins to epithelium development (yellow nodes in Figure 3B), seven proteins to morphogenesis of an epithelium (bright violet nodes in Figure 3B), ten to tissue development (dark green nodes in Figure 4B) and six were proteins of stem cell differentiation (orange nodes in Figure 3B). The significant involved gene ontology (GO) terms are listed for each biological process detected in Supporting Information 2 (Table 5). Finally, the f‐LSCs were cultured in the traditional epithelial differentiation medium (EDM) up to 15 days and we subsequently analysed the molecular profile compared to the control f‐LSCs and LESCs by qPCR analysis (Figure 3C). An increased expression for all corneal epithelial progenitor genes was detected in EDM‐treated f‐LSCs when compared to control f‐LSCs (the negative control for epithelial markers) and interestingly the mRNA expression levels were comparable with the LESC ones (the positive control for epithelial markers). Specifically, no significant changes in pax‐6, ΔNp63α, CK15 (cytokeratin‐15, specific progenitor epithelial markers) and sox17 expression were found (0.65 ± 0.13, 0.44 ± 0.06, 0.51 ± 0.18 FC vs LESCs, respectively, *P* > 0.05). EDM incubation induced remarkable down‐expression of the MSC genes (0.12 and 0.44 FC, *P* < 0.001, for integrin‐β1 and pfn1 mRNA respectively in EDM‐treated f‐LSCs vs the control f‐LSCs). Interestingly, pfn1 mRNA levels were closely comparable with those of LESCs. To verify the mature corneal differentiation capacity of human f‐LSCs, we investigated the expression of epithelial corneal cytokeratin, CK3 and CK12, the two principles corneal lineage markers.^37^ The qPCR analysis was performed on f‐LSCs comparing three different time points of differentiation treatment: 48 hours, 7 and 15 days. A significant time‐dependent increment of CK3 and CK12 mRNA level expression (*P* < 0.024 and *P* < 0.017, respectively) in EDM‐treated f‐LSCs was found (Figure 3D,E).

### 3.4 Knock‐down of profilin‐1 inhibits f‐LSC proliferation and promotes the epithelial genotype: Integrin‐β1, profilin‐1 and nanog a possible central node in f‐LSCs regulation of stemness

The computational analysis suggested involvement of pfn1 in cell fate; to elucidate this role, pfn1 was knocked down. After 72 hours of incubation, pfn1 siRNA‐transfected f‐LSCs showed lower cellular density compared to the control f‐LSCs (Figure 4A: i, iv of the immunofluorescence panel, respectively). Immunofluorescence assays for integrin‐β1 and NANOG were assessed (Figure 4A): in pfn1 siRNA‐transfected f‐LSCs the integrin‐β1 detection was weakly preserved vs the control f‐LSCs (Figure 4A.ii vs 4A.v), whereas we detected no positive stain for NANOG in pfn1 siRNA‐transfected f‐LSCs vs the control f‐LSCs (Figure 4A.viii vs 4A.xi). The lower cellular density evaluated in pfn1 siRNA‐transfected f‐LSCs was investigated by a proliferation curve analysis: the MTT showed that pfn1 siRNA‐transfection significantly inhibited f‐LSC growth when compared to control f‐LSCs at 48, 72 and 96 hours (*P* < 0.05) (Figure 4B). In addition, cell cycle was assessed by flow cytometry to determine the mechanism underlying the observed cell growth inhibition (Supporting Information 2, Figure 3). We evaluated a lower PI in pfn1 siRNA‐transfected f‐LSCs vs the control f‐LSCs (8.75 ± 3.64% vs 26.17 ± 5.12% PI, Figure 4C). Given the overlapping trend of the cell cycle progression between pfn1 siRNA‐transfected f‐LSCs and the nanog siRNA‐transfected f‐LSCs, we analysed the mRNA levels of the stem transcriptional triad and the epithelial related genes in pfn1 siRNA‐transfected f‐LSCs. All f‐LSC stem cell marker expression levels underwent remarkable variations, ie we detected significant up‐regulation of oct4 and sox2 mRNA levels counteracted by a drop in nanog expression when compared to the control f‐LSCs (Figure 4D, upper panel); by contrast, sox17, pax6 and ΔNp63α mRNA were detected (Figure 4D, lower panel). Therefore, these results confirm that pfn1 could have an important role in promotion of f‐LSCs epithelial commitment.

### 3.5 Profilin‐1 acts through mTOR activity

**Figure 5:**
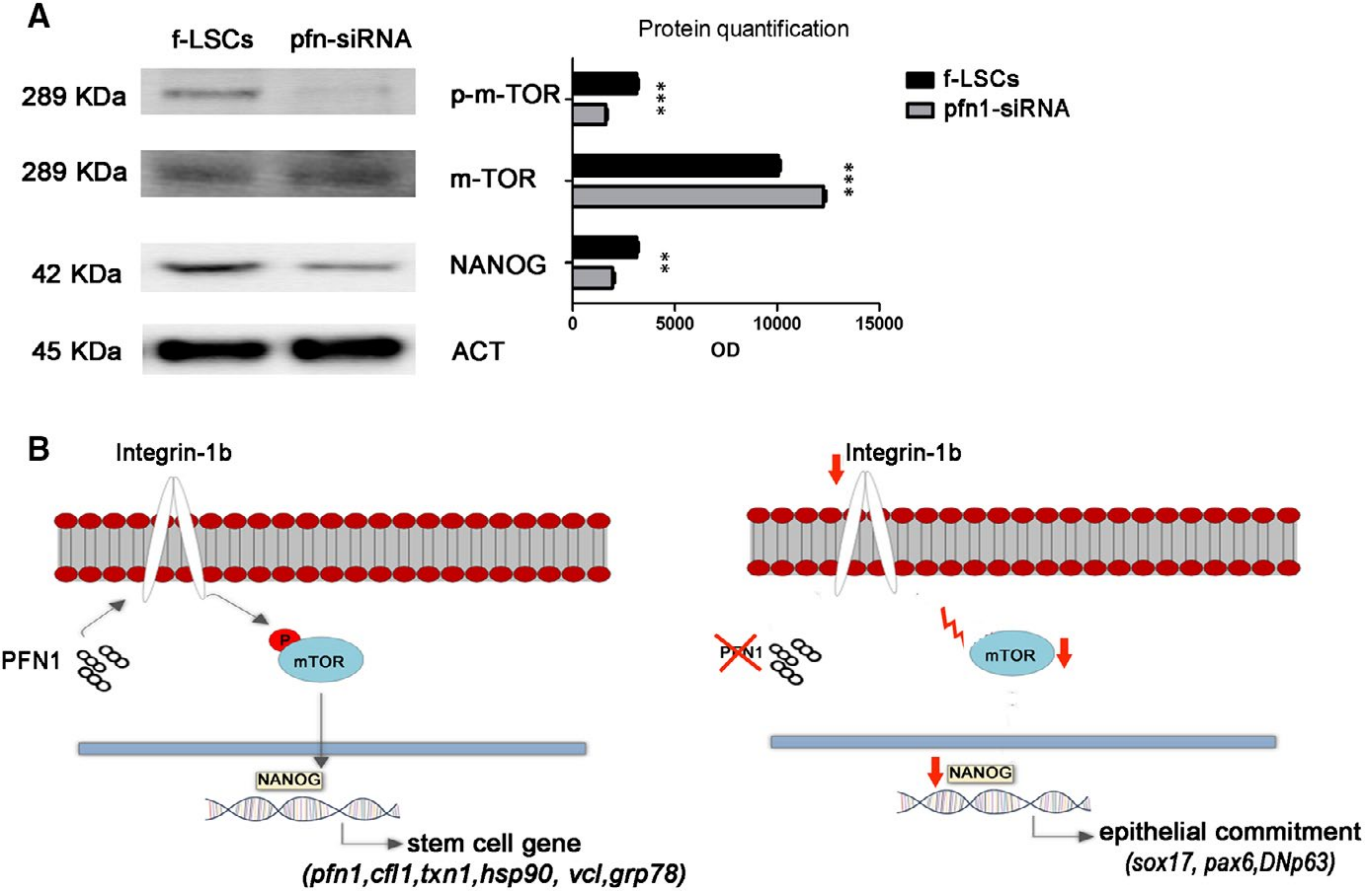
Profilin‐1/mTOR crosstalk. A, Western blot analysis: on the left image are reported the gel bands of P‐m‐TOR, m‐TOR and NANOG; on the right image the histogram graph represents the protein quantification as the optical density (OD) values. B, The schematic diagram of the mechanism underlines the involvement of pfn1, integrin‐β1 and mTOR in regulation of molecular expression. The stem cell gene induction signalling in pfn1 + f‐LSCs (left image), the down regulation of profilin‐1 expression leads f‐LSCs to choose a differentiation fate (right image). mTOR: Mammalian target of rapamycine; P: Phosphate

Because of the closer relation revealed between pfn1 and integrin‐β1 and the closer crosstalk between integrin and mTOR signalling, a Western blot analysis to investigate mTOR activity was performed (Figure 5A). Protein quantification (Figure 5A, right panel) showed that the mTOR activity was down‐regulated up‐to 62.2% in pfn1 si‐RNA f‐LSCs vs the control f‐LSCs. Moreover, we detected a decrease in the NANOG protein level of about 50.8% in siRNA‐transfected pfn1 when compared to the control f‐LSCs. In Figure 5B we show a graphic representation of the mechanism proposed according to our finding. It can be seen that pfn1 and integrin‐β1 co‐operation mediates the activation of the mTOR pathway leading to the transcription of mesenchymal stem genes; whereas when pfn1 was down‐regulated the signalling is inhibited, because of mTOR phosphorylation failure, NANOG decreases its protein levels and switches its partner to SOX17, driving f‐LSCs to epithelial differentiation.

### 3.6 Resveratrol up‐regulates differentiation gene expressions

**Figure 6:**
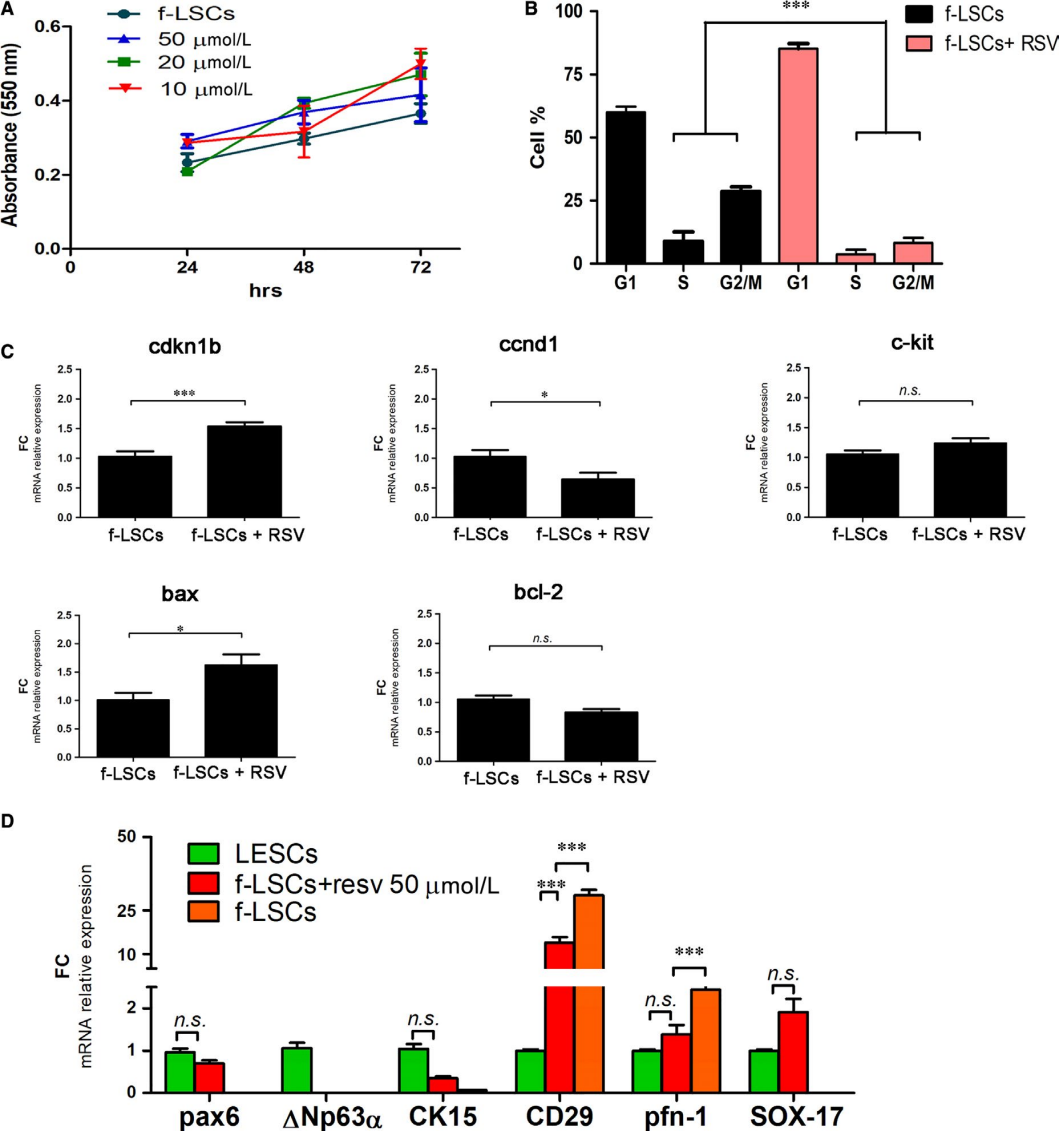
Resveratrol promotes stem/differentiation switch. A, Proliferation assay (MTT) of different RSV concentration‐treated f‐LSCs (10 µM, 20 µM and 50 µM RSV) and the control f‐LSCs. B, The bar plot represents the comparative cell cycle distribution analysis between no‐treated f‐LSCs and RSV‐treated f‐LSCs, at 72 h of treatment; RSV = Resveratrol. C, qPCR analysis of cell cycle regulators and proliferation markers cdnk1b, ccnd1, c‐kit, bax and bcl‐2 in no‐treated f‐LSCs and RSV‐treated f‐LSCs, at 72 h silencing. No‐target siRNA f‐LSCs were used as negative control. D, The bar graphs represent the relative mRNA expression levels of epithelial progenitor markers, pax6, ΔNp63α, CK15, sox17, limbal stromal mesenchymal, CD29 and pfn1 in RSV‐treated/f‐LSCs, no treated f‐LSCs and LESCs. (CD29 = integrin‐β1). The bar graphs (C‐D) were performed by GraphPad Software, Inc, California and are represented as ± SD with *P < 0.05, **P < 0.02, ***P < 0.001, *ns* = no significant, SD = Standard deviation; *P* = *P* value, FC = fold change. All pictures are representative of three independent experiments

Firstly, to exclude an unsafe effect of the RSV in f‐LSCs, an MTT cell viability assay was performed with 10 µM, 20 µM and 50 µM of RSV treatment at 24, 48 and 72 hours. The treatment showed no cell toxicity up to a concentration of 50µM (Supporting Information 3, Figure 4A). At 50 µM of RSV treatment, the growth curve of the treated f‐LSCs kept a linear trend comparable with the control f‐LSC one, except for a weak inflection observed at 72 hours (Figure 6A). Therefore, 50 µM of RSV was chosen for the subsequent experiments: after 72 hours treatment cell cycle and molecular profile analyses were performed. The RSV‐treated f‐LSCs showed a lower cell population in S‐phase when compared to the untreated f‐LSCs (Supporting Information 3, Figure 4B) and we quantified an 8.65 ± 2.17 PI vs a 27.15 ± 6.43 PI respectively for the treated f‐LSCs and the untreated f‐LSCs (Figure 6B). The qPCR analysis for several cell cycle progression regulator genes reflected the cell cycle distribution: a faint variation in ccnd1, cdkn1 and bax mRNA expression levels was counterbalanced by absence of variation in c‐kit and bcl‐2 (Figure 6C). Finally, in the treated f‐LSCs lower levels of integrin‐β1 and up‐regulation of pax6, ck15 and sox17 were detected (Figure 6D). Moreover, we observed an RSV dose‐dependent effect: RSV‐induced regulation of the mRNA expression levels was remarkable at a higher RSV concentration (50 µM vs 10 µM RSV): that is, sox2 and oct4 mRNA levels were up‐regulated, whereas nanog and pfn1 were down‐regulated (Supporting Information 3, Figure 4C).

## 4 DISCUSSION

Nowadays, much greater knowledge of the molecular mechanism underlying the regulation of corneal epithelial specific genes is becoming the major aim in the field of ocular regenerative medicine. It is known that the differentiation process is closely related to expression of the *stem triad* (sox2/oct4/nanog) and its role during commitment processes. Despite the progress in experimental and laboratory procedures, all consecutive steps within the process remain unclear and it is imperative for scientists to have a detailed picture of the peculiar moment when a cell makes the decision to commit itself. In this context, our research focused on profilin‐1 and its relation with the stem transcriptional triad in cell fate determination of f‐LSCs. Previously, we proposed a protein stem‐cell profile enriched with several cytoskeleton‐remodelling and wound healing proteins, including profilin‐1. In this work, we speculate that different expression levels of profilin‐1 could affect the equilibrium between stem and epithelial differentiation‐related genes. Moreover, we show the possibility to modulate profilin‐1 levels with RSV treatment and to obtain an epithelial commitment through down‐regulation of nanog expression. It is known that sox2 and oct4 closely collaborate to maintain higher nanog level expression to ensure cellular self‐renewal.^38^ First of all, we indicate a hierarchy within the *stem triad*: our silencing experiments elect nanog as the key regulator of stemness for its ability to down‐regulate the expression of the f‐LSC stem profile. When a cell chooses differentiation rather than renewal, a balance of bax/bcl‐2 and cdkn‐1/c‐kit slows down the cell cycle 39: our findings highlight this equilibrium in nanog‐siRNA transfected f‐LSCs. Moreover, oct4 replaces sox2, its natural partner, with sox17; nanog is present at lower protein levels and together with all analysed proteins (integrin‐β1, profilin‐1, cofilin‐1, lectin‐1, thioredoxin‐1 and hsp90, pax6 and ΔNp63α, bax, ccnd1 and cdkn1b) is one of the protagonists of a differentiation network.^40^, ^41^, ^42^, ^43^, ^44^ The f‐LSCs appeared to be a ΔNp63α‐/SOX17‐ and highly integrin‐β1+/PFN1+/NANOG + cell population. By contrast, when pfn1 is knocked down both integrin‐β1 and nanog are reduced and the cells show a lower rate of growth in spite of the appearance of epithelial related genes, as revealed by cell cycle and proliferation‐related gene analyses.^45^, ^46^ Finally, we investigated the effect of RSV on f‐LSCs. It is known that RVS and other polyphenols have beneficial effects on several anterior eye cells‐based diseases and promote differentiation in vitro culture.^47^, ^48^, ^49^, ^50^ Our findings show that RSV treatment does not affect f‐LSCs viability, whereas it outlines a limbal epithelial progenitor‐like molecular profile characterised by expression of sox17, pax6, ΔNp63α, CK15 and emphasises involvement of profilin‐1, integrin‐β1 and nanog. Cheng et al proposed a mechanism underlying inhibition of gastric cancer progression through profilin‐1 silencing: they demonstrated that profilin‐1 silencing inhibits gastric cancer cell proliferation, migration and invasion through the integrin‐β1 pathway, with involvement of mTOR activity.^51^ We speculate that a similar mechanism could occur in f‐LSCs during epithelial differentiation and a working model of differentiation process regulation could be developed: as suggested by our findings, pfn1 affects intracellular events downstream of integrin‐β1/mTOR including epithelial gene expression.

In summary, most ocular surface diseases are caused by dysfunction of LSCs, which leads to the inability to preserve corneal epithelial physiology. To the best of our knowledge, this is the first work proposing a possible differentiation of f‐LSCs into limbal epithelial progenitor‐like cell by resveratrol‐induced profilin‐1‐down‐regulation. Moreover, the supplying of factors in vivo able to protect and activate f‐LCS could be the linchpin to identify a resolving treatment of corneal diseases with limbal stem cell dysfunction. Our results suggest that profilin‐1 and its related functions may be considered excellent candidates for development of a successful cell‐based therapy to resolve ocular damaged supporting regeneration and wound healing.

## ETHICS APPROVAL AND CONSENT TO PARTICIPATE

The study was approved by the Ethical Committee of the AOUP, University of Palermo (No. 09/2009). Human tissues were used in accordance with the Declaration of Helsinki and informed written consent was given by each patient. This study was supported by the Università degli Studi di Palermo (PON01_00829: Innovative Technology platforms for tissue engineering).

## CONFLICTS OF INTEREST

The authors declare that they have applied for two patents concerning the possible use of fibroblast‐like limbal stem cells in type 1 diabetes (PCT/EP2010/070860) and autoimmunity diseases (PCT/EP2017/082380). We declare that none of the co‐authors have any non‐financial competing interests in relation to this manuscript.

## AUTHOR CONTRIBUTIONS

LT: Conception and design, Collection and/or assembly of data, data analysis and interpretation, manuscript writing. AC, MP, GP: Data analysis and interpretation. VF and CS: Provision of material or patients. CG: Provision of study material or patients, financial support, Final approval of manuscript.
