# Supplementary material for: PFN1 and integrin‐β1/mTOR axis involvement in cornea differentiation of fibroblast limbal stem cells
Source: J Cell Mol Med. 2019 Sep 12;23(11):7210–21. doi: 10.1111/jcmm.14438 (PMC6815913; doi:10.1111/jcmm.14438)
Supplement: Supplementary file 1 [file JCMM-23-7210-s001.pdf]

Supporting Information 1.

Supporting material informations.

PPFN1 and Integrin-β1/mTOR axis involvement in cornea differentiation of fibroblast limbal stem cells

Laura Tomasello[a], Antonina Coppola[a], Maria Pitrone[a], Valentina Failla[b], Salvatore Cillino[b], Giuseppe Pizzolanti[*,a], Carla Giordano[*,a]

**Sirna (h)**	**Code product**	**Concentration, incubation.**
Oct-4	sc-43980, (Santa Cruz Biotechnology, Inc)	50nM; 72 hrs
Sox-2	sc-38408,(Santa Cruz Biotechnology, Inc)	50nM; 72 hrs
Nanog	sc-43958, (Santa Cruz Biotechnology, Inc)	50nM; 72 hrs
Profilin-1	sc-36316, (Santa Cruz Biotechnology, Inc)	50nM; 72 hrs

Table 1. siRNA (h) duplex oligoribonucleotides used gene silencing in f-LSCs.

GENE	PRIMER SEQUENCE	CODE NUMBER
NANOG		QT01844808
OCT3/4		QT00210840
SOX2	F- GGAGACGGAGCTGAAGCCGC<br>R-GACGCGGTCCGGGCTGTTTT	MWG
Profilin-1	F:5'-ACCCGGAAACAAGAAGAC-3'<br>F:5'-ACTGGTCCGATAACCTCCCA-3'	MWG
Cofilin-1	F:5'-TGCGGCTCCTACTAAACGG-3'<br>F:5'-ACGCACCTTCATGTCGTTGA-3'	MWG
Vinculin	F:5'-ATGTCTCCTATATCCTGGTTT-3'<br>F:5'-GCAGGAAGTGTCCTTCAGAC-3'	MWG
grp-78	F:5'-TACAGCAGATGGTGAGCGAC-3'<br>R:5'-TGCTGTGTGCCCCAAGTAAT-3'	MWG
lectin-5	F:5'-CCACAGCCAGTACCCAACTT-3'<br>R:5'-TTTTGCCCCCACCCAAGAAT-3'	MWG
ΔNp63α	F:5'-GAGGTTGGGCTGTTCATCAT-3'<br>R:5'-GTGGGAAAGAGATGGTCTGG-3'	Eurofins genomics
Sox-17		QT90204099
Integrin-β1 (CD29)		QT00068124
Pax-6		QT00071169
CK3		QT00050365
CK15	F:5'-GGAGGTGGAAGCCGAAGTAT-3'<br>R:5'-GAGAGGAGACCACCATCGCC 3'	
ccnd1		QT00495285
cdkn1b		QT00998445
c-kit		QT01679993
bcl-2		QT00997416
bax		QT00997381

Table 2. The qRT-PCR primers used for gene expression investigation.

Antibody, localization marker	Code number	Dilution	Incubation	Assay
**Primary antibody**				
SOX17	SantaCruz, sc-20099	1:50	Over/night	FC
ΔNp63α	SantaCruz, sc-8344	1:10	Over/night	FC
Integrin-β1 (CD29), surface	Milteny Biotec, 130-101-258	1:10	30 min	FC,IF
NANOG, intracellular	Milteny Biotec, 130-105-050	1:50	30 min	FC,IF
NANOG, intracellular	SantaCruz, sc-293121	1:100	Over/night	WB
Profilin-1, intracellular	Abcam, ab124904	1:10	30 min	FC,IF, WB
**Secondary antibody**				
AlexaFluor 488 (FITC)	LifeTechnologies, Z25402	1:50	20 min	
AlexaFluor 594 (PE)	Life Technologies, Z25007	1:50	20 min	

Table 3 Human monoclonal antibodies used in immunofluorescence staining (IF), flow cytometry (FC) and western blot analysis (WB).

1. Analysis of Cell Cycle Progression. For DNA content analysis Nicoletti's protocol was performed. Briefly, 1x106 cells were fixed in 70% ethanol, rehydrated in PBS and then re-suspended in a DNA extraction buffer (PBS with 0.2 M $NaHPO_4$, 0.1% Tritonx-100, pH 7.8). After staining with 1 μg/mL of propidium iodide (PI) for 5 minutes the samples were acquired with FACS Calibur flow cytometer (Becton-Dickinson, New Jersey, USA) and fluorescence was defined by analysis performed with CellQuest (Becton Dickinson) software. The percentages of G1, S, and G2 phase cells were calculated with the MODFIT-LT software program (Verity Software House, Inc.). The Proliferation Index percentage (PI) was expressed as the sum between the percentage of cell in G2 and M-phase.
