# Supplementary material for: PFN1 and integrin‐β1/mTOR axis involvement in cornea differentiation of fibroblast limbal stem cells
Source: J Cell Mol Med. 2019 Sep 12;23(11):7210–21. doi: 10.1111/jcmm.14438 (PMC6815913; doi:10.1111/jcmm.14438)
Supplement: Supplementary file 2 [file JCMM-23-7210-s002.pdf]

Supporting Information 2.

Supporting siRNA data.

PFN1 and Integrin-β1/mTOR axis involvement in cornea differentiation of fibroblast limbal stem cells

Laura Tomasello[a], Antonina Coppola[a], Maria Pitrone[a], Valentina Failla[b], Salvatore Cillino[b], Giuseppe Pizzolanti[*,a], Carla Giordano[*,a]

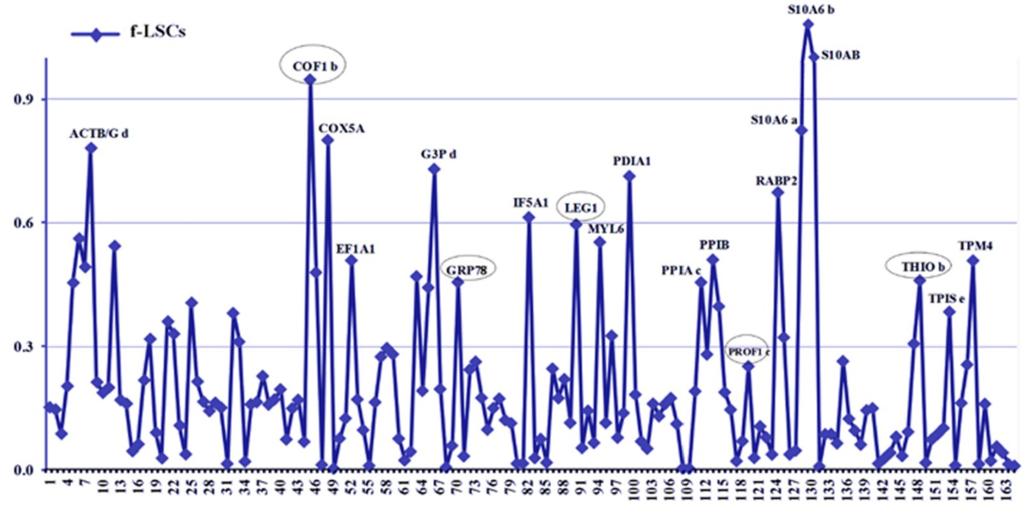

Figura 1: The stem proteomic profile in f-LSC. Related to fig1.

siRNA/ f-LSCs	f-LSC STEM CELL MARKERS						
	pfn1	cofilin	vinculin	grp78	lectin-1	txn	hsp90
oct-4	0.49 ± 0.09	0.29 ± 0.021	4.32 ± 0.28	1.21 ± 0.07	0.22 ± 0.06	1.43± 0.21	1.02 ± 0.15
sox-2	0.29 ± 0.07	0.05 ± 0.014	6.37 ± 0.18	0.93 ± 0.04	0.87 ± 0.12	-	0.07 ± 0.07
nanog	0.06 ±0.01	0.19 ± 0.02	8.99 ± 0.05	0.47 ± 0.03	0.87± 0.21	3.5 ± 0.84	1.97 ± 0.5

Table 1 Calculated fold change values of f-LSC genes in gene silencing experiments.

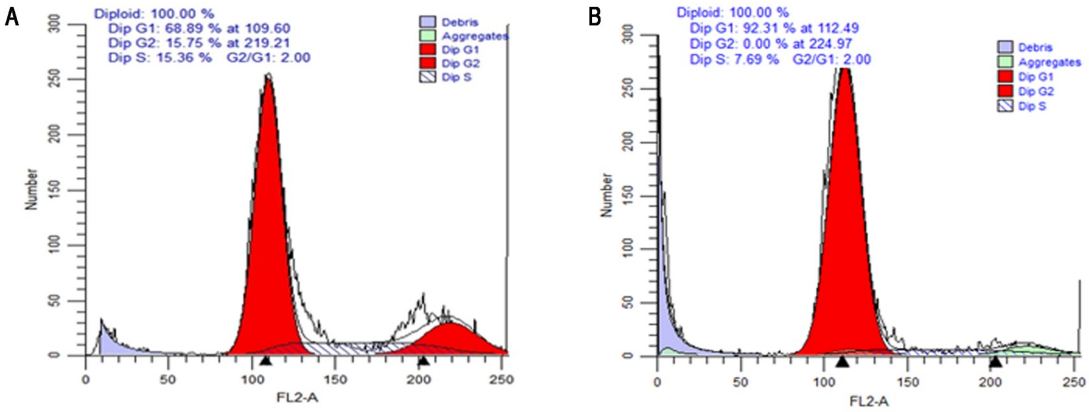

Figure 2. The nanog-silencing arrests the cell cycle progration. Related to Figure 2A.

pathway ID	pathway description	count in gene set (node)	FDR
GO:0009611	response to wounding	10	4.78E-07
GO:0001708	cell fate specification	5	8.93E-06
GO:0001775	cell activation	8	1.9E-05
GO:0060429	epithelium development	9	2.17E-05
GO:0002009	morphogenesis of an epithelium	7	3.13E-05
GO:0009888	tissue development	10	3.6E-05
GO:0042127	regulation of epithalial cell proliferation	6	4.65E-05
GO:0048863	stem cell differentiation	6	7.13E-05

Table 2 Functional annotation terms using DAVID tool.

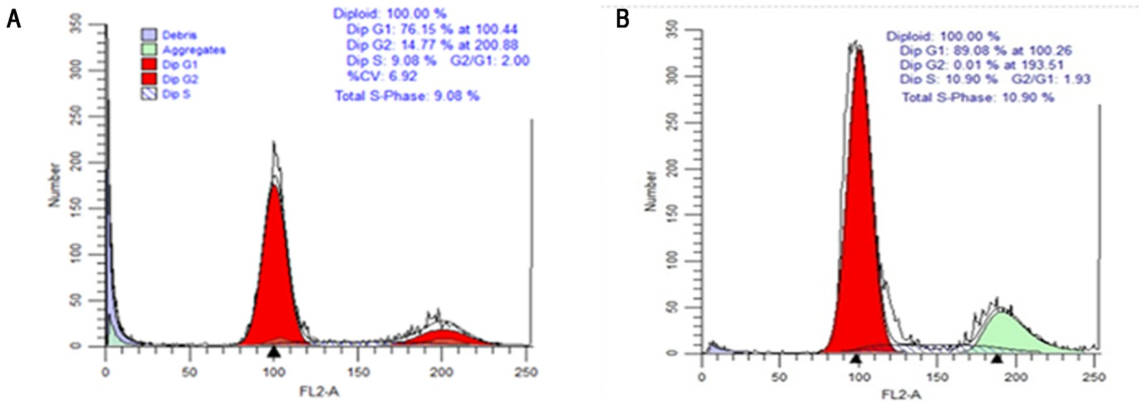

Figura 3: The profilin-1 silencing arrests the cell cycle progression. Related to Figure 4C.
