# Supplementary material for: PFN1 and integrin‐β1/mTOR axis involvement in cornea differentiation of fibroblast limbal stem cells
Source: J Cell Mol Med. 2019 Sep 12;23(11):7210–21. doi: 10.1111/jcmm.14438 (PMC6815913; doi:10.1111/jcmm.14438)
Supplement: Supplementary file 3 [file JCMM-23-7210-s003.pdf]

Supporting Information 3.

Supporting data.

PFN1 and Integrin-β1/mTOR axis involvement in cornea differentiation of fibroblast limbal stem cells

Laura Tomasello[a], Antonina Coppola[a], Maria Pitrone[a], Valentina Failla[b], Salvatore Cillino[b], Giuseppe Pizzolanti[*,a], Carla Giordano[*,a]

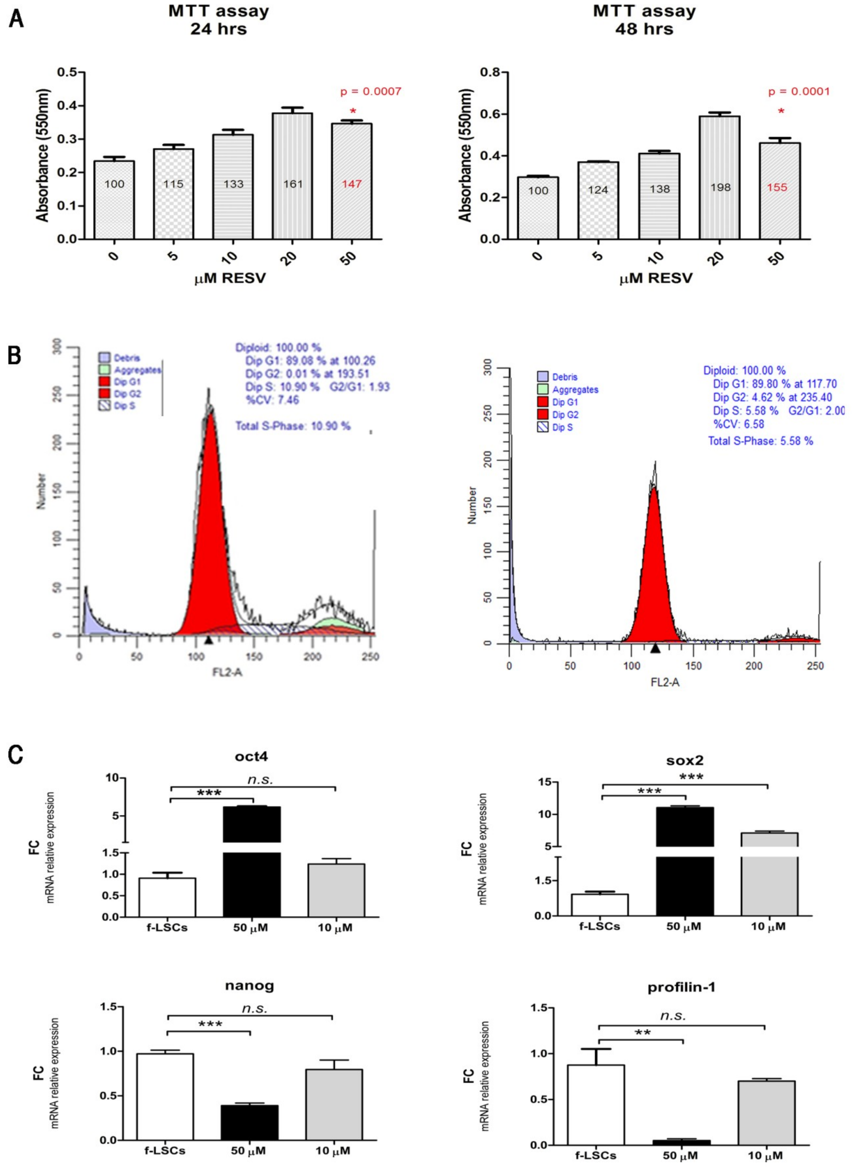

Figure 1. The Resveratrol treatment mimes pfn1-silencing. Related to Figure 5.
